# Gene expression of PMP22 is an independent prognostic factor for disease-free and overall survival in breast cancer patients

**DOI:** 10.1186/1471-2407-10-682

**Published:** 2010-12-15

**Authors:** Dan Tong, Georg Heinze, Dietmar Pils, Andrea Wolf, Christian F Singer, Nicole Concin, Gerda Hofstetter, Ingrid Schiebel, Margaretha Rudas, Robert Zeillinger

**Affiliations:** 1 Department of Obstetrics and Gynaecology, Medical University of Vienna, EBO 5Q, AKH, Währinger Gürtel 18-20, Vienna 1090, Austria; 2 Section of Clinical Biometrics, Center for Medical Statistics, Informatics and Intelligent Systems, Medical University of Vienna, Spitalgasse 23, Vienna 1090, Austria; 3 Ludwig-Boltzmann Cluster Translational Oncology; EBO 5Q, AKH, Waehringer Guertel 18-20, Vienna 1090, Austria; 4 Department of Gynaecology and Obstetrics, Innsbruck Medical University, Christoph-Probst-Platz, Innrain 52, Innsbruck 6020, Austria; 5 Clinic Institute of Pathology, Medical University of Vienna, Währinger Gürtel 18-20, Vienna 1090, Austria

## Abstract

**Background:**

Gene expression of peripheral myelin protein 22 (*PMP22*) and the epithelial membrane proteins (*EMPs*) was found to be differentially expressed in invasive and non-invasive breast cell lines in a previous study. We want to evaluate the prognostic impact of the expression of these genes on breast cancer.

**Methods:**

In a retrospective multicenter study, gene expression of *PMP22 *and the *EMPs *was measured in 249 primary breast tumors by real-time PCR. Results were statistically analyzed together with clinical data.

**Results:**

In univariable Cox regression analyses PMP22 and the EMPs were not associated with disease-free survival or tumor-related mortality. However, multivariable Cox regression revealed that patients with higher than median *PMP22 *gene expression have a 3.47 times higher risk to die of cancer compared to patients with equal values on clinical covariables but lower *PMP22 *expression. They also have a 1.77 times higher risk to relapse than those with lower *PMP22 *expression. The proportion of explained variation in overall survival due to *PMP22 *gene expression was 6.5% and thus PMP22 contributes equally to prognosis of overall survival as nodal status and estrogen receptor status. Cross validation demonstrates that 5-years survival rates can be refined by incorporating *PMP22 *into the prediction model.

**Conclusions:**

*PMP22 *gene expression is a novel independent prognostic factor for disease-free survival and overall survival for breast cancer patients. Including it into a model with established prognostic factors will increase the accuracy of prognosis.

## Background

Breast cancer is by far the most frequent cancer of women with about one million new cases every year worldwide. Even though the prognosis for breast cancer patients is rather good, it is still the leading cause of cancer mortality in women causing about 400,000 annual deaths [1]. So far, the most important prognostic factor is lymph node status, which indicates disease-free survival and overall survival in breast cancer. The well defined predictors include the presence of hormone receptors that predict the response to endocrine therapy and the HER2 status that predicts the response to Tratuzumab. However, there is no predictive factor for chemotherapy that can be clinically used [2]. The prognosis of breast cancer is also far from being precise. Identification of new prognostic and predictive markers will not only help patients to receive the proper treatment, it can also provide new therapeutic targets.

The invasive potential of tumor cells reflects the intrinsic characteristics of tumor cells. Genes involved in the invasive process might therefore correlate with outcome of the disease and have certain prognostic and predictive values. In a previous study, we characterized the cell lines derived from breast cancer or normal breast tissues by their invasive ability to penetrate into a collagen-fibroblast matrix and compared gene expression profiles of invasive and non-invasive cell lines using Affymetrix GeneChip technology [3]. Several genes, which had not been described in the context of breast cancer, were identified and validated by RT-PCR. Two of these genes code for members of a subfamily of small hydrophobic membrane proteins, namely EMP3 and PMP22. Both are highly expressed in most of the invasive cell lines and had very low expression levels in non-invasive cell lines.

The whole family consists of the peripheral myelin protein 22 (PMP22) and the epithelial membrane proteins (EMP1, -2, and -3), which are expressed in many tissues, and have functions in cell growth, differentiation, and apoptosis [4].

We hypothesize that these genes can have prognostic impacts on breast cancer. The objectives of the study are defined as the measurement of the expression of the *EMPs *and *PMP22 *in tumor tissues from 249 breast cancer patients using real-time RT-PCR, statistical evaluation of their prognostic impacts, and assessment of their added values to already established prognostic factors.

## Methods

### Breast cancer patients

This is a retrospective study, including 249 sporadic primary breast cancer patients from the Department of Obstetrics and Gynecology, Medical University of Vienna, Vienna; and the Department of Gynecology and Obstetrics, Innsbruck Medical University, Innsbruck (Table 1) from 1987 to 2001. All procedures were approved by the institutional advisory boards. The age of the patients ranged from 27 to 89 years with a mean age of 58 and a median age of 57 at the time of diagnosis. All patients underwent a close follow-up scheme consisting of regular visits with a complete physical examination. Ultra-sound examination of the abdomen and chest X-ray were performed every 6 months. Mammography and bone scan were performed every 12 months or in cases of suspect clinical findings. There were 109 cases of recurrence in the study. Clinical endpoint of recurrent disease was proven histologically or otherwise indicated by X-ray, computer tomography, or bone scan as measurable disease. Patients who did not die from cancer were censored at the date of death. There were 71 cases of death within the observation time. The follow-up period ended in 2008 with a median follow-up time of 85 months.

**Table 1:** Patients' age and histopathological characteristics of tumors

		Mean gene expression			
		
	Sample number (%)	*EMP1*	*EMP2*	*EMP3*	*PMP22*
	249 (100.0)	1.090	0.225	0.115	0.491
**Age**					
≤50 years	84 (33.7)	1.108	0.236	0.113	0.500
>50 years	165 (66.3)	1.081	0.220	0.117	0.487

**Histological type**					
invasive ductal carcinoma	189 (75.9)	1.045	0.216	0.110	0.482
invasive lobular carcinoma	40 (16.1)	1.385	0.280	0.133	0.558
others and unknown	20 (8.0)	1.006	0.220	0.133	0.455

**Nodal status**					
pN0	95 (38.1)	1.128	0.224	0.122	0.524
pN1	124 (49.8)	1.059	0.228	0.110	0.467
unknown	30 (12.1)	1.102	0.217	0.117	0.493

**Tumor size**					
pT1 (<2 cm)	65 (26.1)	1.153	0.226	0.125	0.528
pT2 (2-5 cm)	127 (51.0)	1.011	0.199	0.110	0.453
pT3 (>5 cm)	23 (9.2)	1.024	0.289	0.111	0.514
pT4	14 (5.6)	1.378	0.395	0.127	0.590
others and unknown	20 (8.0)	1.331	0.246	0.116	0.543

**Differentiation grade**			**p = 0.005**		**p = 0.019**
G1	34 (13.7)	1.194	0.351	0.147	0.713
G2	123 (49.4)	1.131	0.250	0.116	0.531
G3	71 (28.5)	0.978	0.165	0.100	0.357
unknown	21 (8.4)	1.096	0.171	0.120	0.497

**Estrogen Receptor**		**P < 0.0001**	**p < 0.001**	**p < 0.001**	**p < 0.001**
positive	133 (53.4)	1.325	0.344	0.141	0.715
negative	116 (46.6)	0.875	0.138	0.092	0.319

### Specimen characteristics

Fresh tumor biopsies from breast carcinomas were collected during surgery and snap frozen immediately after the histologic examination of frozen sections. Only samples consisting of at least 90% tumor tissues were collected. Clinical pathological parameters were determined at the Department of Pathology, Medical University of Vienna. Characteristics of tumors are shown in Table 1. Tumor biopsies were frozen in liquid nitrogen until further processed.

### Total RNA preparation

Tissues were homogenized using a microdismembrator and dissolved in GI lysis buffer (4 M Guanidine Isothiocyanate, 0.5% N-lauroyl-Sarcosine, 10 mM EDTA, 5 mM Sodium Citrate, and 100 μM β-mercaptoethanol). Total RNA was extracted from tumor biopsy lysates by isopycnic centrifugation as described previously [5] followed by a DNA digestion step of incubation with RNase-free DNase I (Roche Diagnostic, Mannheim, Germany) at 37°C for 15 minutes. The quality of the RNA was examined with RNA 6000 Nano Chips and RNA 6000 Nano Reagent & Supplies on a 2100 Bioanalyzer (Agilent Technologies, Waldbronn, Germany). RNA concentrations were determined spectrophotometrically.

### Reverse transcription (RT)

RT was carried out using Omniscript Reverse Transcriptase kit (Qiagen, Hilden, Germany). The total reaction volume was 20 μl including 500 ng RNA. The reaction mixture was incubated at 37°C for 60 min, heated at 95°C for 10 min and then cooled on ice. The reaction was diluted 1:4 with water and aliquoted for further analysis.

### Real-time PCR

The primers and probes for beta-2-microglobulin were included in TaqMan PDAR B2 M RNA Control Reagent (Applied Biosystems, Foster City, CA). For the quantifications of *EMPs*, *PMP22 *and *ER*, "Assay-on-Demand" kits (Applied Biosystems) were used (*ER*: Hs00174860_m1; *EMP1*: Hs00608055_m1; *EMP2: *Hs00171315_m1; *EMP3*: Hs00171319_m1 and *PMP22*: Hs00165556_m1). 5700 Sequence Detection System (Applied Biosystems) was used for real-time analysis. 4 μl diluted cDNA aliquot was used as template for PCR in a total volume of 25 μl including TaqMan Universal PCR Master Mix and the corresponding probes and primers. The mixture was pre-incubated at 95°C for 10 min followed by 40 cycles of two step incubations at 95°C for 15 s and 60°C for 1 min. All samples were measured in duplicates.

### Quantitation of gene expression

The relative quantitation method with standard curves was used for the calculation of the relative amounts of mRNAs. A sample with a high expression level of a certain gene was chosen as calibrator. Its expression was defined as 1. A standard curve using serial dilutions of the calibrator was used to calculate the amount of RNAs in other samples. Target quantities of all other samples were expressed as n-fold in relation to the calibrator. To correct the quantity differences in the starting RNA samples, the target quantity of certain mRNA was normalized to that of the constitutively expressed house keeping gene beta-2-microglobulin in the same sample.

### Estrogen receptor status by expression analysis

Protein levels of estrogen receptor (ER) in tumors were primarily determined using immunohistochemistry. Since ER status was missing for 56 samples (22.5%), we re-determined it using mRNA gene expression values. A similar procedure was suggested and used in a previous study [6]. We measured the ER gene expression in a cohort of breast cancer tissues with known clinical ER status obtained by immunohistochemistry and used that value of ER gene expression as cutoff point for ER status, which minimized the sum of false positive and false negative rates.

### Statistical analysis

For model building, the following parameters were considered besides the expression values of the markers analysed: age at diagnosis, histological type, nodal status, tumor size, differentiation grade, and estrogen receptor status.

Mean values and 95% confidence intervals for genes expression were calculated on a logarithmic scale (log2) and then transformed back to the original scale (Table 1). In order to compare the gene expression between two or more groups, T-test or one-way ANOVA, respectively, were performed using the log-transformed expression as independent variable with subsequent Bonferroni-Holm correction for multiple testing.

Disease-free survival is defined as time between diagnosis of disease and recurrence or distant metastasis. Overall survival is defined as time from diagnosis of disease to death of patients of breast cancer. Patients who died of causes unrelated to breast cancer were treated as censored in disease-specific survival analysis. Median follow-up time was computed by the Kaplan-Meier method with reverse status indicator as proposed by Schemper and Smith [7]. For analysis of disease-free and overall survival, tumors with differentiation grade 1 and 2 were combined for comparison with those with differentiation grade 3 and tumors with pT1 were compared with those combining pT2, pT3 and pT4. These groupings were necessary because of the low number of cases in some subgroups.

The assumptions of the Cox models (additivity of effects, proportional hazards, linearity of effects) were assessed as follows. First, interactions of pairs of variables were evaluated by including and testing corresponding product terms. Second, time-dependency of hazard ratios was accounted for by testing correlation of scaled Schoenfeld residuals with time [8]. Third, gene expression was entered into analysis by using the log2-values instead of categories, and a potential non-linear effect of gene-expression was tested by including additional model terms that were derived from a linear-tail restricted cubic spline. These sensitivity analyses uncovered a time-dependent effect of estrogen receptor status on tumor-specific survival, which was accounted for by including a time-dependent covariate defined as the product of estrogen receptor status (1 or 0) and the logarithm of survival time. However, this did not alter any conclusion on the other variables.

The predictive ability of the multivariable models was assessed by computing the proportion of explained variation due to Schemper and Henderson [9]. Furthermore, relative importance of variables was assessed by omitting variables one-by-one from the multivariable model as suggested by Heinze and Schemper [10]. Furthermore, we evaluated the predictive ability using ten-fold cross-validation as follows: first, the data set was randomly split into 10 approximately equally-sized subsamples. Second, nine of the ten subsamples were merged to form a training sample. Regression coefficients were estimated for the multivariable model including all variables using the training sample, and risk scores were predicted for the remaining tenth subsample (the test sample). These risk scores were obtained by inserting the estimated regression coefficients and the observed variable values of the test sample into the linear model equation. This second step was repeated ten times in turn such that each subject once appeared in the test sample and such was assigned a cross-validated risk score. Third, the cross-validated risk scores were stratified into quartiles and Kaplan-Meier curves, 5-year survival rates and a log-rank test were used to describe the association of risk scores with survival. The process was repeated, omitting gene expression variables from the model to assess if and to which extent prediction worsens if gene-expression was not accounted for. This ten-fold cross-validation was seen as more appropriate than a single split-up into a training set and a test set, as the former yields more accurate results than the latter [11]. P-values < 0.05 were considered as indicating statistical significance. The statistical software packages R 2.4.2 http://www.r-project.org and SAS 9.1.3 (2003 SAS Institute Inc., Cary, NC) were used for statistical graphics and analyses, respectively.

## Results

Estrogen receptor status was determined as follows: Using 207 samples, of which we had both, immunohistochemical and gene expression results of ER, we determined a cut-off value of 0.4984 for the gene expression to differentiate ER positive and ER negative tumors. Using this value to judge the status of ER, the original immunohistochemical data had a 19.2% false positive and 26.5% false negative rate, respectively. All samples in this study were re-evaluated for their ER status using this cut-off value, which generated 116 negative and 133 positive tumors.

Expression data of *EMP1*, *EMP2*, *EMP3 *and *PMP22 *were obtained from all tumor samples. After log2 transformation, the data presented normal distribution (Table 1). The age of the patients at diagnosis (median: 58 yrs, range 27 - 89 yrs) and clinical data, including tumor size (pT), differentiation grade (G), nodal status (pN), and histological type of the tumors were analyzed for possible correlations with expression of *EMPs *and *PMP22 *(Table 1). Statistical analysis revealed a significant inverse correlation of *EMP2 *and *PMP22 *expression in tumor tissues with differentiation grade (p = 0.005 and p = 0.019, respectively). In addition, all gene expressions showed significant direct correlation with positive ER status. Expressions of *EMP1*, *EMP2 *and *EMP3 *had no prognostic values either for disease-free survival (DFS) or overall survival (OS) (Table 2 and 3). However, expression of *PMP22 *was a strong prognostic factor for outcome in a multivariable Cox regression model. Patients with higher than median *PMP22 *gene expression had a 3.47 times higher risk to die of cancer than patients with lower than median *PMP22 *expression (Table 2). They also had a 1.77 times higher risk to relapse than those with lower than median *PMP22 *expression levels (Table 3). These hazard ratio estimates were adjusted by tumor size, ER, nodal status, differentiation grade, and age of the patients (age only for overall survival). Results did not change materially if an interaction of ER status with log of survival time was added to the model in order to account for non-proportional hazards of ER status (data not shown). No significant interaction of the effect of PMP22 with any other variables in the model was detected. Analysing log PMP22 levels instead of PMP22 categories did not improve the model fit, neither of the univariate nor of the multivariable models. Furthermore, an exploratory analysis evaluating as potential cut-off values all deciles of PMP22 did not improve over the categorization at the median PMP22 value.

**Table 2:** Estimates of hazard ratios for tumor related-death

	Univariate Analysis			Multivariable Analysis		
Variable*	**Hazard Ratio**	**95% Confidence Interval Of Hazard Ratio**	**p**	**Hazard Ratio**	**95% Confidence Interval of Hazard Ratio**	**p**

*EMP1*	1.11	0.69 - 1.77	0.6664			
*EMP2*	0.82	0.59 -1.51	0.8223			
*EMP3*	1.11	0.69 - 1.77	0.6703			
*PMP22*	1.29	0.81 - 2.07	0.2851	3.47	1.82 - 6.62	**0.0002**
pT	1.58	1.10 - 2.26	**0.0125**	1.68	1.15 - 2.47	**0.0078**
pN	3.68	2.00 - 6.77	**<0.0001**	3.46	1.80 - 6.65	**0.0002**
G	1.03	0.72 - 1.47	0.8887	1.06	0.70 - 1.62	0.7741
Age	0.52	0.33 - 0.84	**0.0069**	0.58	0.34 - 0.98	**0.0406**
ER	0.53	0.33 - 0.84	**0.0077**	0.31	0.17 - 0.55	**<0.0001**

*The hazard ratios are indicated for: *EMP1*, *EMP2*, *EMP3*, and *PMP22 *(> median gene expression) vs. (≤ median gene expression);

(pT3+pT4) vs. (pT1+pT2); pN1 vs. pN0; (G2+G3) vs. G1; Age > 50 vs. Age ≤ 50; ER+ vs. ER

**Table 3:** Estimates of hazard ratios for recurrent disease

	Univariate Analysis			Multivariable Analysis		
Variable*	**Hazard Ratio**	**95% Confidence Interval of Hazard Ratio**	**p**	**Hazard Ratio**	**95% Confidence interval of Hazard Ratio**	**p**

*EMP1*	1.01	0.69 - 1.47	0.9738			
*EMP2*	1.03	0.70 - 1.50	0.8930			
*EMP3*	0.93	0.64 - 1.36	0.7275			
*PMP22*	1.01	0.69 - 1.46	0.9780	1.77	1.09 - 2.87	**0.0200**
pT	1.32	0.99 - 1.78	0.0610	1.27	0.93 - 1.74	0.1367
pN	2.72	1.73 - 4.27	**< 0.0001**	2.72	1.69 - 4.38	**< 0.0001**
G	1.06	0.78 - 1.44	0.7033	0.98	0.70 - 1.38	0.9133
ER	0.63	0.43 - 0.92	**0.0169**	0.49	0.31 - 0.78	**0.0025**

*The hazard ratios are indicated for: *EMP1*, *EMP2*, *EMP3*, and *PMP22 *(> median gene expression) vs. (≤median gene expression);

(pT3+pT4) vs. (pT1+pT2); pN1 vs. pN0; (G2+G3) vs. G1; ER+ vs. ER-

The proportion of variation in time to tumor-specific death explained by *PMP22 *expression and all covariates was 22.3%. This value dropped by 6.5%, 6.7% and 6.7%, if *PMP22 *expression, pN or ER status were left out from the multivariable model, respectively (Table 4). This result suggests that these three variables are equally important to predict mortality of breast cancer. In case of DFS, pN, ER status, and gene expression of *PMP22 *account for 13.0% prediction. If pN, ER, and *PMP22 *gene expression was omitted from the model, than the prediction will reduced by 7.9%, 4.0%, and 2.3%, respectively, indicating the most contribution of pN for DFS (Table 4).

**Table 4:** Proportion of explained variation (PEV)

	Disease-free survival	Overall survival
PEV	Partial (%)	Partial (%)
pN	7.9	6.7
ER	4.0	6.7
*PMP22*	2.3	6.5
pT	1.0	2.8
Age	-	1.5
G	0.0	0.1

**Figure 1:**
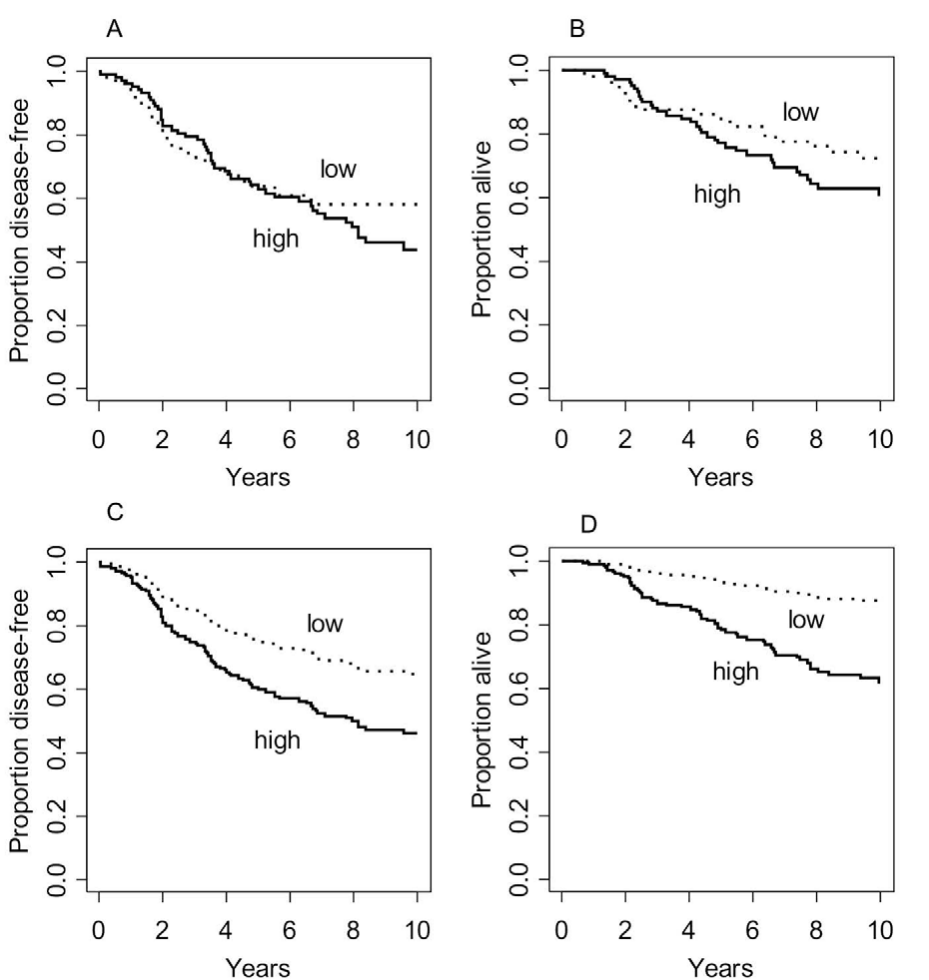
**Kaplan-Meier curves comparing patients with high and low *PMP22 *gene expression (dichotomized at the median)**. 1A. DFS, not adjusted; 1B. OS, not adjusted; 1C. DFS, adjusted survival function from Cox model (comparing high and low *PMP22 *gene expression for patients with average values for T, pN, G, and ER); 1 D. OS, adjusted survivor function from Cox model (comparing high and low *PMP22 *gene expression for patients with average values for T, pN, G, ER, and age).

No association of *PMP22 *gene expression with OS and DFS was seen in unadjusted analyses using Kaplan-Meier curves (Figure 1). The additional prognostic value of *PMP22 *in multivariable models must therefore be attributed to certain correlation of *PMP22 *expression with other strong predictors. Indeed, *PMP22 *expression is positively correlated with ER status (Table 1 p < 0.0001). Thus the effects of *PMP22 *expression and ER status were compromised in unadjusted analyses. Similar but weaker associations exist between *PMP22 *gene expression and prognostic values of tumor size and lymph node involvement. Therefore we have computed adjusted survival curves from the multivariable Cox models, which refer to the estimated survival of patients with mean values on tumor size, lymph node involvement, differentiation grade, age and ER expression, and high or low *PMP22 *expression (Figure 1).

Cross validation of the 5-year survival rates showed that the model including *PMP22 *expression has a broader range of prediction, therefore a better discrimination of different risk groups than models excluding *PMP22 *expression. This is true for both DFS and OS (Table 5), showing that PMP22 has an additive value in predicting survival after the diagnosis of breast cancer.

**Table 5:** 5-year survival rates calculated by cross validation

	5-year DFS rate		5-year OS rate	
**Quartile (Risk Scores)**	**with *PMP22***	**without *PMP22***	**with *PMP22***	**without *PMP22***

1 (lowest risk)	88%	87%	98%	91%
2 (intermediate low risk)	63%	68%	87%	91%
3 (intermediate high risk)	59%	48%	74%	78%
4 (highest risk)	41%	47%	62%	59%

Rate difference (lowest to highest risk)	47%	40%	36%	32%

	**p = 0.001**		**p<0.0001**	

## Discussion

*PMP22 *and *EMPs *were selected for the evaluation of their prognostic values based on their higher expression levels in invasive breast cell lines compared to non-invasive ones. The invasiveness of these cell lines was determined by the ability of the cells to penetrate into a collagen-fibroblast matrix [12]. Cell motility and the capacity to invade into the surrounding tissues are preconditions for tumor cells to metastasize. Genes that are not expressed or expressed to less extent in non-invasive cells but are highly activated in invasive cells could be markers for prediction of tumor metastases. They could also indirectly indicate the outcome of patients. Indeed, we showed that patients with higher expression of *PMP22 *in their tumors have both, worse DFS and OS, suggesting that PMP22 is involved directly or indirectly in the invasion process. Our study also suggests that invasive and non-invasive cell lines provide a useful model for searching for prognostic factors.

In this study, we did not only show that *PMP22 *gene expression has prognostic value on DFS and OS, we also showed that PMP22 gene expression is as powerful as nodal status and ER status to predict mortality of breast cancer patients by calculating the proportion of explained variation. Traditionally, the prognostic values of gene expression were only evaluated by multivariable Cox regression model. The gain of including additional prognostic factors was not well addressed. Even though many new prognostic biomarkers have been reported, quite often they don't increase the predictive accuracy when added to the established clinical predictive factors [13]. By leaving out one of the three important prognostic factors, namely *PMP22 *gene expression, pN, or ER, the proportion of explained variations decreased equally, demonstrating that *PMP22 *expression contributes equally to prognosis as pN or ER status does. Therefore, PMP22 has potential use in clinical practice.

Using cross validation, we compared the ranges of 5-year survival rates of breast cancer patients between models including and excluding *PMP22 *gene expression. In these analyses, risk scores were stratified into quartiles. The results show that by including *PMP22 *gene expression into the prediction model, the accuracy of the prediction was significantly increased. This indicates that including of *PMP22 *gene expression into clinical risk evaluation can refine the prognosis, again showing the added value of *PMP22 *gene expression to prognosis. It is of great interests to establish a prognostic score including *PMP22 *gene expression values and other know independent factors.

So far, expression and functions of PMP22 have been well investigated in neuroscience. Abnormalities in PMP22 can lead to various peripheral neuropathies [14]. Increased PMP22 expression was found in other pre-malignant or malignant tissues, like pancreatic tissues [15], osteosarcoma, and glioblastoma tissues [16,17]. However, little is known about the functions of PMP22 in human cancer. It is very interesting to further investigate the possible functions of PMP22 in breast cancer and to clarify its roles in tumor invasion, so that we can better understand its prognostic impact.

## Conclusions

In this study we show that breast cancer patients with higher than median *PMP22 *gene expression had a 3.47 times higher risk to die of cancer than patients with lower than median *PMP22 *expression. They also had a 1.77 times higher risk to relapse than those with lower than median *PMP22 *expression levels. The analysis of the proportion of explained variation suggests that gene expression of *PMP22*, ER status and pN variables are equally important to predict mortality of breast cancer. Cross validation of the 5-year survival rates showed that the model including *PMP22 *expression has a broader range of prediction, therefore a better discrimination of different risk groups than models excluding *PMP22 *expression. This is true for both DFS and OS, showing that PMP22 has an additive value in predicting survival after the diagnosis of breast cancer.

Taking together, *PMP22 *gene expression is an independent prognostic factor for disease-free and overall survival for breast cancer patients. It contributes equally to the prediction of cancer related death as estrogen receptor status and nodal status. Including *PMP22 *gene expression into a multivariable model including ER and nodal status, the accuracy of the prediction can be increased.

Functional studies on PMP22 in breast cancer should be investigated to elucidate its roles in the progression of breast cancer and to explain if it could be a therapy target.

## Competing interests

The authors declare that they have no competing interests.

## Authors' contributions

DT participated in the design and coordination of the study and drafted the manuscript; DP and GH participated in the design of the study, performed the statistical analysis, and helped to draft the manuscript; CS, NC and GH participated in the design of the study and contributed to the acquisition of clinical data; AW and IS performed most of the experiments; MR is responsible for the histopathological reports and RZ participated in the design and conception of the study and have given the final approval. All author read and approved the final manuscript.

## Pre-publication history

The pre-publication history for this paper can be accessed here:

http://www.biomedcentral.com/1471-2407/10/682/prepub
